# Artificial Vegetation for Sand Stabilization May Impact Sand Lake Dynamics in Dune Regions

**DOI:** 10.3390/plants13020255

**Published:** 2024-01-16

**Authors:** Alamusa, Yuhang Su, Quanlai Zhou, Zhiyu Liu, Yongcui Wang, Xiao Zheng

**Affiliations:** 1 CAS Key Laboratory of Forest Ecology and Management, Institute of Applied Ecology, Chinese Academy of Sciences, Shenyang 110016, China; alamusa@iae.ac.cn (A.); suyuhang21@mails.ucas.ac.cn (Y.S.); zhouquanlai@126.com (Q.Z.); 2 University of Chinese Academy of Sciences, Beijing 100049, China; 3 College of Journalism and Communications, Bohai University, Jinzhou 121000, China; bobo66w@163.com

**Keywords:** sand dunes, sand-fixing vegetation, sand lakes, water transport

## Abstract

Vegetation on dunes regulates the water supply from the dunes to the inter-dune lowland, which is a crucial factor affecting lake water dynamics in the inter-dune lowland. Previous researchers have paid insufficient attention to the water regulation function of dunes on a landscape- and regional scale. To fill this gap, both remote sensing technology and field observations were used to analyze the variations in the lake area and their influence factors, such as vegetation coverage and precipitation in the lake watershed, on a multi-year scale (2000–2020) and one-year scale (2021), respectively. The results showed that precipitation is the main factor influencing the changes in lake water, and artificial sand vegetation can regulate the changes in lake water. On the multi-year scale, with the coverage of artificial sand-fixing vegetation increasing on sand dunes in the lake watershed, the areas of the lakes were gradually decreasing. On the one-year scale, with dune vegetation coverage increased, the water supply from dunes to lakes showed a decreasing trend. This model can provide a possibility for estimating and predicting the influence of water supply from dunes to lakes that is affected by sand-fixing vegetation. The findings have significant theoretical and practical utility for the rational utilization of water resources in sandy land, as well as for assisting in the selection of an optimized construction mode for desert control projects.

## 1. Introduction

There is a close hydraulic connection between dunes and inter-dune lowlands in sandy land ecosystems [1,2]. Sand dunes’ soil water can be transferred to the inter-dune lowland through deep seepage and lateral flow processes, making the dunes a primary “water reservoir” in sandy lands [3]. The soil water is initially recharged into groundwater through vertical migration, which then connects with adjacent sand lakes through horizontal transportation [4].

Vegetation on dunes plays a crucial role in regulating the water migration between dunes and inter-dune lowlands [1,5]. Sand-fixing vegetation significantly restricts soil water movement within the dunes, changes the proportion of each component of the water balance in sandy ecosystems, and reduces the water that is supplied from dunes to inter-dune lowlands [3,6]. The study conducted by Zhao et al. demonstrated that artificial vegetation could increase the heterogeneity of the soil water distribution both horizontally and vertically [7]. Li et al.’s study revealed that plants utilized a 40 cm to 300 cm depth of soil moisture, because the deep soil moisture content decreased significantly [8]. Kizito et al.’s investigation indicated that shrubs consumed soil water effectively, resulting in a limited water supply [9].

Human intervention played a dominant role in altering the dune mobility of most dunes during the past century [10]. The alteration of sand dune vegetation induces the modification of the environmental factors surrounding the dunes [11,12,13]. Since the 1960s, numerous desert control projects have been implemented on sandy land in China. Through establishing a higher coverage of artificial sand-fixing vegetation, vast expanses of active sand dunes have been converted into stabilized sand dunes, which has mitigated the hazards from sandstorms effectively [8]. A majority adhere to a minimum vegetation coverage standard of 40% in these large-scale sand-fixing afforestation initiatives [14]. However, the encroachment of vegetation could degrade the soil moisture in dunes, limit water percolation into deeper layers, and weaken the hydraulic connection between dunes and surrounding landscape units [15]. This could result in a water imbalance in the dune ecosystem, leading to a reduction in or even drying up of the sand lake water [16]. The alteration of the hydrological cycle, as a consequence of sand-fixing vegetation, is one of the primary driving forces behind this water imbalance in the region [17,18]. Chang et al. discovered that with the sand-fixing vegetation coverage being increased, the area of sand lakes decreased by 26% in Horqin Sandy Land, China [19].

Because of this problem, previous research has paid more attention to the impacts of sand-fixing vegetation on soil water on the individual- and community scales, but insufficient attention has been paid to the water regulation function of dunes on the landscape- and regional scales [20,21,22,23,24,25]. There are various explanations for the decrease in inter-dune lowland water (sand lakes) in sandy areas. These include reduced precipitation as a result of climate change, human overuse of water resources, and increased vegetation coverage in sandy regions [26,27].

This paper focuses on the effects of large-scale artificial sand-fixing vegetation on sand lakes on the watershed scale. Both remote sensing technology and field observations were used to analyze the variations in the lake area and their influencing factors in Horqin Sandy Land, China, such as vegetation coverage and precipitation in the lake watershed, on a multi-year scale and one-year scale, respectively. We investigated the impact of artificial sand-fixing vegetation on lake areas. The findings have significant theoretical and practical meaningfulness for the rational utilization of water resources in sandy land, as well as for assisting in the selection of an optimized construction mode for desert control projects.

## 2. Materials and Methods

### 2.1. Study Area

**Figure 1:**
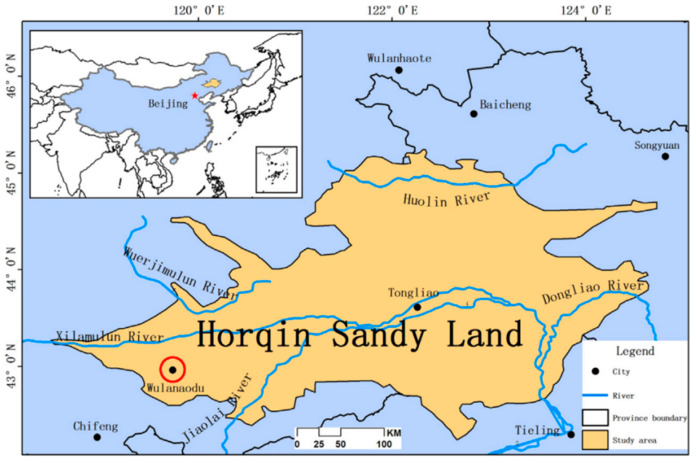
The location of the study area in the Horqin Sandy land, China.

**Figure 2:**
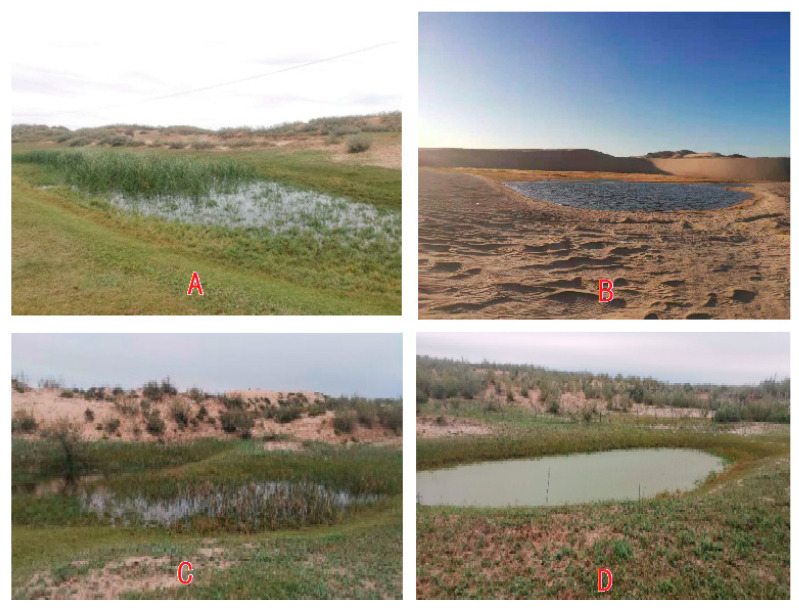
The typical look of sand and sand lake landscapes in Horqin Sandy Land. (**A**) A stabilized dune and sand lake; (**B**) active dune and sand lake; (**C**) semi-active dune and sand lake; (**D**) semi-active dune and sand lake.

The study site is situated in Horqin Sandy Land, which is located in northern China (Figure 1). The region has a typical temperate continental monsoon climate with low humidity and a fluctuating distribution of annual precipitation. The annual average precipitation stands at 284.4 ± 82.4 mm, with more than 70% being received between June and August, while the precipitation during spring (March to May) constitutes merely 10% of the total annual precipitation. The mean daily evaporation in the region is 5.33 ± 0.79 mm, with a coefficient of variation of 14.77% [21]. Within the study area, dunes and inter-dune lowlands are found adjacent to each other. Typically, inter-dune lowlands have a watershed with different areas, and sand lakes can sometimes be observed in these inter-dune lowlands. The primary habitat types in the study area include active dunes, stabilized dunes, and inter-dune lowlands and sand lakes (Figure 2). The stabilized dunes have been established by sand barriers and the planting of shrub plants, such as *Caragana microphylla* Lam. and *Hedysarum fruticosum* Pall.

### 2.2. Research Methods

Both remote sensing technology and field observations were used to analyze the variations in the lake area and their influencing factors, such as vegetation coverage and precipitation in the lake watershed, on a multi-year scale (2000–2020) and one-year scale (2021), respectively. On the multi-year scale, we aimed to analyze the relationship between the lake area and dune vegetation coverage within its surrounding watershed. Meanwhile, on the one-year scale, we focused on assessing the influence of dune vegetation coverage on the water supply from dunes to lakes. The research methods are described below in terms of the multi-year scale and one-year scale.

#### 2.2.1. Research Methods on the Multi-Year Scale

We collected remote sensing data and historical climate data from the years 2000 to 2020. Landsat and Modis remote sensing images were utilized to gather information on the lake’s area and dune vegetation coverage over the years. Meteorological data were sourced from the Ulanaodu Desertification Control Experimental Station, which is adjacent to the study area.

##### The Method of Plot Setting

After conducting field investigations and analyzing historical data, three inter-dune lowland watersheds containing sand lakes in Horqin Sandy Land were selected as the research subjects. They are the HuitunTala (H), Xigaorisu (X), and Nailinggao (N) plots. Each watershed plot consists of three landscape types: lake, inter-dune lowland, and dune.

During the study period, the Nailinggao plot was significantly affected by human interference, which was unsuitable for this study’s needs. Consequently, the Nailinggao plot was excluded, with only the Huituntala and Xigaorisu plots remaining for further examination.

##### Methods for Determining the Watershed Area and Its Composition

**Figure 3:**
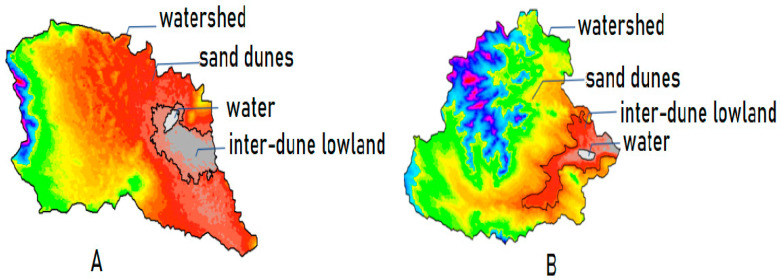
Demonstration of the method of dividing the watershed and its content within the plots. (**A**) Huituntala plot; (**B**) Xigaorisu plot.

We applied the Digital Elevation Model (DEM) elevation data classification method to determine the watershed region, dune region, and inter-dune lowland region for each plot. We analyzed the DEM data using ArcGIS10.6 software and segmented the watershed area, inter-dune lowland area, dune area, and lake area of each plot by utilizing the watershed module (Figure 3). The results are displayed in Table 1.

##### Methods for Determining the Dune Vegetation Coverage of the Watershed

The MOD13Q1 dataset from MODIS was utilized to acquire vegetation coverage information for each plot from 2000 to 2020. The pixel dichotomy model was employed within ENVI5.3 software to execute band operations on the synthesized NDVI data and generate a corresponding vegetation coverage image. Using the vegetation coverage image in conjunction with the plot vector boundary that was previously mentioned, the coverage image of the study plot was extracted via the MASK tool located within the spatial analysis module of ArcGIS10.6 software. Based on the boundary of the dune area, vegetation pixel values were classified, counted, and ultimately utilized to derive vegetation coverage data for various years.

In this study, the vegetation in the inter-dune lowland has always been in a natural state and has not been disturbed by human activities. It is regarded as constant vegetation coverage, and its impact on the hydrologic process remains unchanged. Therefore, this study focuses on the impact of dune vegetation on sand lakes in its watershed.

#### 2.2.2. Research Methods on the One-Year Scale

##### The Method for Categorizing Plots

Based on the vegetation coverage of dunes and the condition of lakes in the inter-dune lowland, we identified the inter-dune lowland watershed with a lake as the research plots in Horqin Sandy Land. The selected plots were categorized into distinct types based on the vegetative coverage of the dunes in the watershed. These included stabilized dune watersheds, semi-active dune watersheds, and active dune watersheds (Figure 2). We selected 2–3 plots of each type as research objects.

##### Method for Determining the Watershed Area and Its Compositions

The watershed range of the plots was delineated by using the first dune ridge around the inter-dune lowland as the boundary. The internal enclosed area was designated as the watershed range, and the watershed area, dune area, and lake area were measured manually using GPS. A total of 8 experimental plots were selected in this study (Table 2). The study plots of the stabilized dune watershed were named T_1_, T_2_, and T_3_, the plots of the semi-active dune watershed were named L_1_ and L_2_, and the plots of the active dune watershed were named Y_1_, Y_2_, and Y_3_.

##### Method for Determining the Lake Water Variation

A water level monitoring device (U20-001-01, HOBO, with an accuracy of ±0.1 cm) was installed in each plot’s lake to constantly monitor water level changes (in 30 min intervals from June to October). GPS was utilized to manually measure the lake area every 15 days from June to October.

##### The Acquisition Method for the Vegetation Coverage of Dunes

Thirty-six vegetation survey quadrants measuring 6 m × 6 m each were randomly placed in the dune area of each plot. The crown width of suffruticosa plants within each quadrant was measured every 30 days throughout the growth season. The vegetation coverage of the quadrants was determined by calculating the percentage ratio of the sum of the projected area of the plants’ crowns to the total area. The mean coverage value of each quadrant was used to determine the coverage of the entire plot. Due to the small size of individual herbaceous plants and their scarcity in the sand dunes, the coverage of herbaceous plants was not included in this study.

##### The Calculation Method for the Water Recharge from the Dune to the Lake

The fluctuation of lake water (ΔS) was primarily impacted by precipitation (R), dune-to-lake recharge water (W), and evaporation (E). We calculated the variation in lake water during a specific time period (the time interval between two consecutive observations, which in this study was 15 days) using water level observation data and water area data. The formula is as follows:

(1) $$△S=\frac{\left(S1+S2+\sqrt{S1\times S2}\right)}{3}\times h$$

where △S is the lake water volume change during the periods; *S*1 and *S*2 are the area of the lake at time 1 and time 2, respectively. *h* is the difference in lake water elevation between time 1 and time 2.

Next, we can accurately determine the amount of water that is supplied from the dune to the lake (W) by analyzing the precipitation and lake surface evaporation during the same timeframe. Precipitation (R) was obtained through field observation methods. The evaporation (E) of the lake surface was calculated using the evaporation parameter calculation method. Because the sandy lake is connected with groundwater, the process of lake leakage is not considered in this study. The rainfall data here come from a self-recording rain gauge set up at the test site; the surface evaporation data are obtained from the self-recording surface evaporation meter set up at the test site. The water balance of the lake is formulated as follows:

W + R = △S + E(2)

Using the above formula, we can obtain the amount of water that is recharged to lakes (W). To accurately quantify the water replenishment capability of dunes to lakes, we performed a standard treatment on the water recharge capacity of the dunes. We utilized the ratio of the total water replenishment to the dune area during the observation period as the dune’s water replenishment capacity per unit area. This methodology enabled us to compare the dune’s water replenishment capacity under different levels of vegetation coverage.

##### Statistical Analysis

Data processing and statistical analysis were conducted using Microsoft Excel 2013 and SPSS 18.0, while Origin 8.5 software was utilized for creating visualizations. We took the water supply of dunes as the dependent variable; took precipitation, dune vegetation coverage, and temperature as independent variables; and adopted the stepwise regression method to further explore the quantitative relationship between each factor.

## 3. Results

### 3.1. The Long-Term Impacts of Sand-Fixing Vegetation on Sand Lakes

#### 3.1.1. The Variation Dynamic of Sand Lake Areas on a Multi-Year Scale

**Figure 4:**
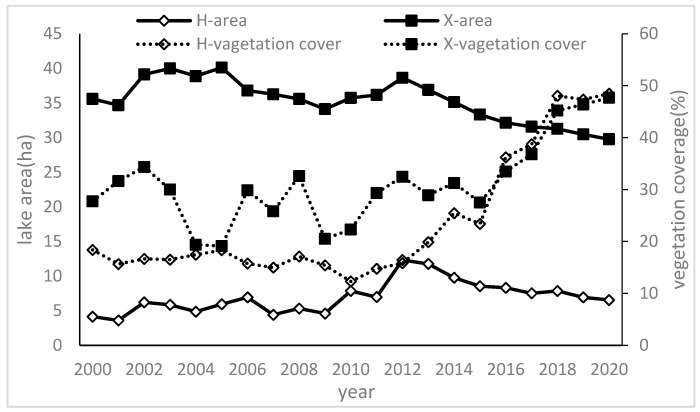
Variation in lake area and dune’s vegetation coverage in the watershed of two plots during 2000–2020. H: Huituntala plot; X: Xigaorisu plot.

The changes in the lake area showed two distinct stages: in the first stage, the lake area showed a fluctuating change from 2000 to 2012, and in the second stage, the lake area showed a gradually decreasing trend from 2013 to 2020. The variation dynamic of the sand lakes in both plots is shown below. The Huituntala plot displayed a fluctuating trend in its lake area between 2000 and 2020 (Figure 4). The lake area showed relatively stable fluctuation from 2000 to 2009, only about 5 ha fluctuation. However, the lake area experienced a rapid increase from 2010, reaching a peak in 2012, and subsequently decreased. For another plot, the size of the sand lake in the Xigaorisu plot showed a decreasing trend initially, followed by a gradual upward trend from 2000 to 2020. In particular, the lake area showed a decreasing trend from 2012, and the lake area had decreased by 1.20 times by 2020.

We found that the Xigaolisu plot initially had a larger area compared to the Huituntala plot. The area of the lake in the Huituntala plot showed significant changes due to its small size, which was more likely to be affected by external factors. The coefficient of variation (CV) of the area change was recorded as 32.46% for the Huitongtala plot and 8.52% for the Xigaolisu plot.

#### 3.1.2. The Variation Characteristics of Dunes’ Vegetation Coverage during 2000–2020

The detailed variation dynamics of both plots are shown below.

In the Huituntala plot, the dune’s vegetation coverage remained consistent, with minimal variation and with a coefficient of variation of 9.74% from 2000 to 2012. Since 2013, the vegetation coverage in the dunes has increased rapidly, from 19.86% in 2013 to 48.45% in 2020 (Figure 4). As for the Xigaorisu plot, the vegetation coverage of the dunes showed small fluctuations, and the coefficient of variation was 18.85% from 2000 to 2012. Since 2013, the dune’s vegetation coverage has increased rapidly, from 28.90% in 2013 to 47.69% in 2020.

An analysis of the changes in the vegetation cover of the dunes in these two plots revealed that there has been an increase in vegetation coverage from 2013 to 2015. This was an important time for the vegetation cover to change. According to historical records of the study area, a large-scale desert control project was initiated between 2013 and 2015. By constructing straw checkerboard barriers and planting shrubs on the active dunes, sand-fixing vegetation with a vegetation coverage over 45% was established within 3–5 years. Therefore, the dune vegetation coverage has shown a rapid increase since 2015.

#### 3.1.3. An Analysis of Factors Influencing the Change in Sand Lake Area on the Multi-Year Scale

**Figure 5:**
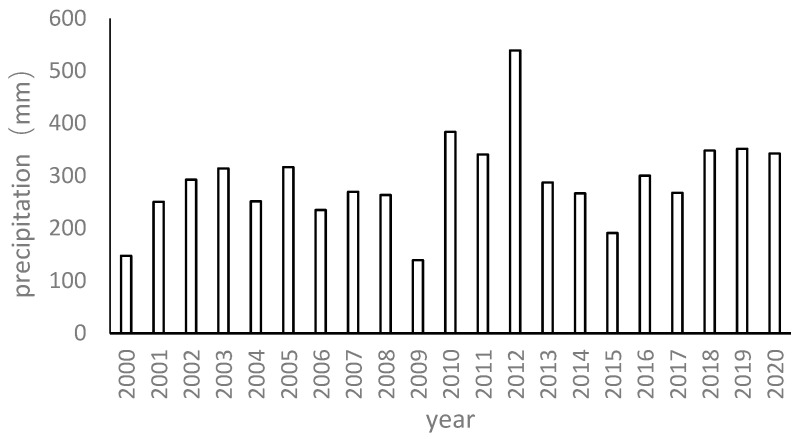
The precipitation data across the 2000–2020 time period.

All plots remained in their natural state from 2000 to 2012. The correlation between the lake area and precipitation during this period revealed that precipitation demonstrated a positive correlation with the lake area, and the correlation coefficients between precipitation and the lake area were found to be 0.876 for the Huituntala plot and 0.451 for the Xigaorisu plot (Figure 5). However, no significant correlation was found between the lake area and the vegetation coverage of sand dunes within the watershed during this period.

Through an analysis of the relationship between the lake area and dune vegetation coverage following the introduction of artificial sand-fixing vegetation in 2013, a significant negative correlation was observed between the lake area and dune vegetation coverage in both the Huituntala and Xigaorisu plots from 2013 to 2020. The correlation coefficients for these relationships were determined to be −0.861 ** and −0.832 **, respectively (the correlation is significant at the 0.01 level).

This study found that the presence of artificial vegetation designed for stabilizing sand dunes had a significant impact on the change in the lake area. As the vegetation coverage and age of plants on the dunes increased, there was a corresponding decrease in the lake area.

### 3.2. The Relationship between Lake Water Variation and Dune Vegetation Coverage on a One-Year Scale

#### 3.2.1. The Variation Characteristics of Lake Areas during the Growing Season

**Figure 6:**
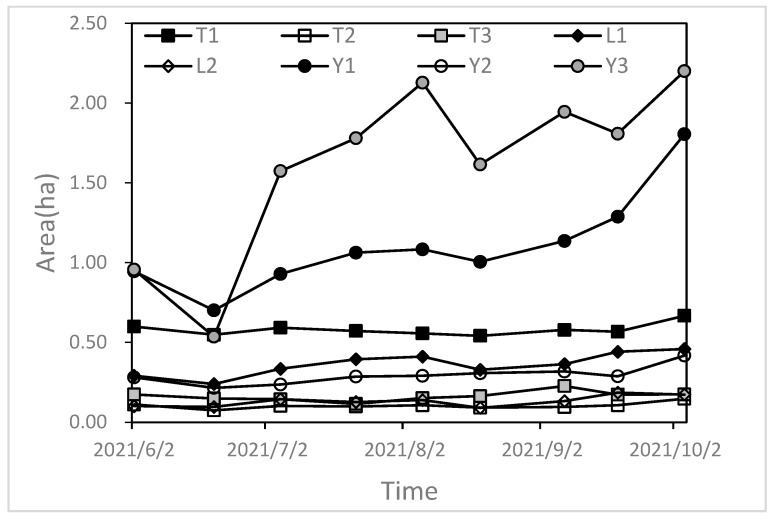
Variation in lake area in the growing season. T_1–3_: lake area of stabilized dune plots; L_1–2_: lake area of semi-active dune plots; Y_1–3_: lake area of active dune plots.

This study indicated that all plots exhibited an expanding trend in the lake area during the growing season (Figure 6). The average area of the lakes in the stabilized dune watershed plots rose by 0.03 ha, with the lowest coefficient of variation (CV) being 14%. In the semi-active dune watershed plots, the mean lake area increased by 0.12 ha, with a CV of 21%. In the active dune watershed plots, the mean lake area rose by 0.75 ha, with the largest CV being 25%. During the growing season, the lake area of the active dune watershed plot had the largest single increase of 1.03 ha, which occurred on 5 July. The single largest increase in lake area was 0.1 ha on 5 July in the semi-active dune watershed plot. The stabilized dune watershed plot had the lowest single increase of 0.04 ha, which occurred on 4 October.

#### 3.2.2. The Dynamic of Dune Vegetation Coverage during the Growing Season

The mean vegetation coverage of dunes for stabilized dune watershed plots, semi-active dune watershed plots, and active dune watershed plots were 35.75%, 23.53%, and 3.30% during the growing season, respectively, with a range of variation in coverage of 27.17–48.68%, 17.69–29.45%, and 0.8–8%.

During the growing season, there was a gradual increase in the dune vegetation coverage across all plots. Stabilized dune watershed plots increased by 9.60%, semi-active dune watershed plots increased by 4.24%, and active dune watershed plots increased by 4.4%. The range of increase varied from 3.20% to 12.42%, with the largest increase occurring between July and August.

#### 3.2.3. The Effect of Dune Vegetation Coverage on the Water Recharge from Dune to Lake

**Figure 7:**
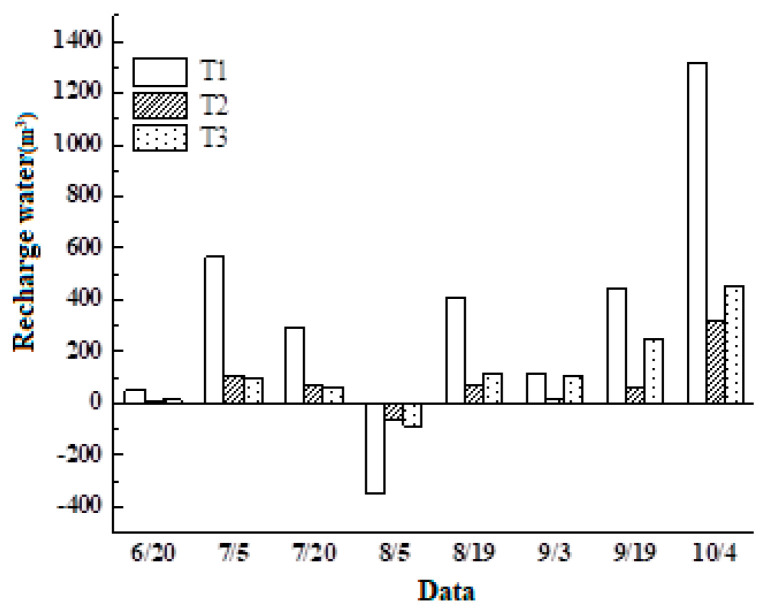

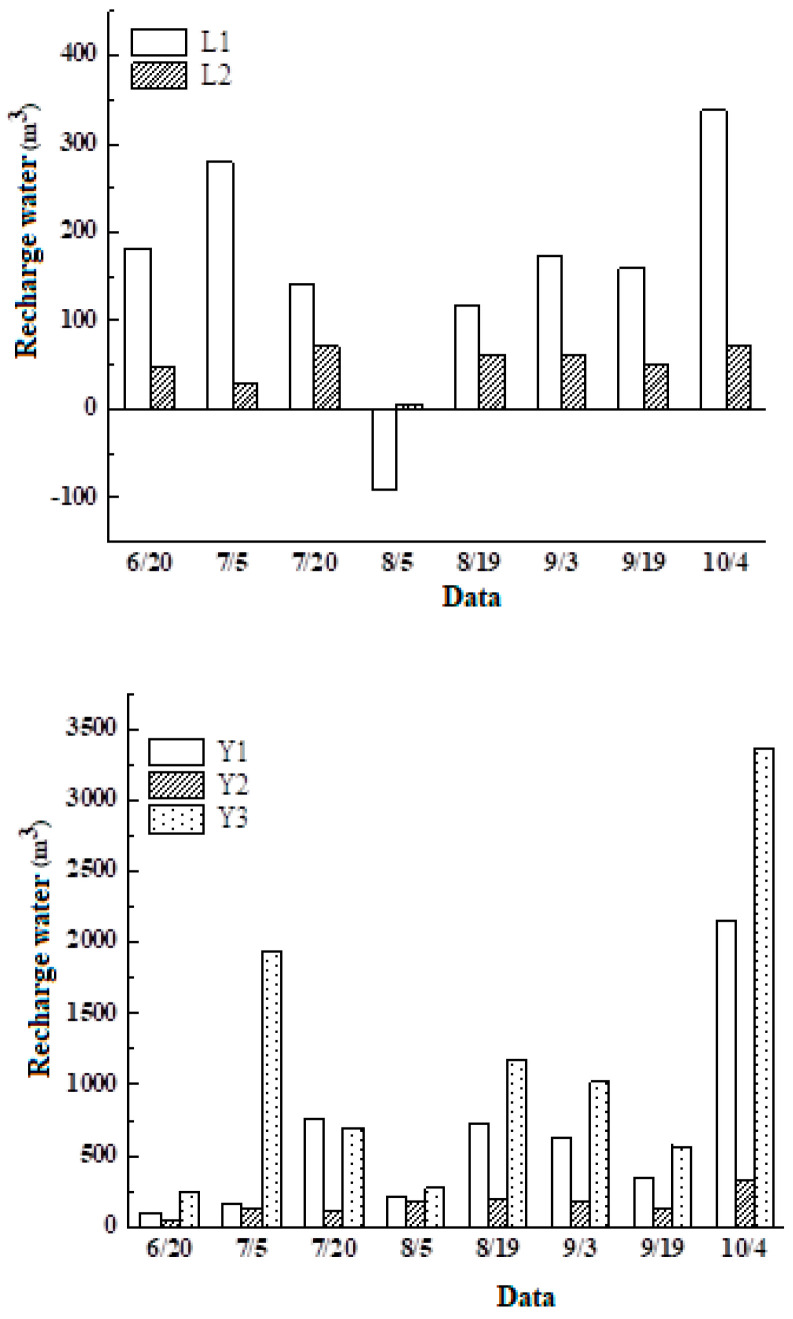
The water is recharged from dunes to lakes during the growing season. T_1–3_: stabilized dune plots; L_1–2_: semi-active dune plots; Y_1–3_: active dune plots.

The water recharged from dunes to lakes varies throughout the growing season (Figure 7). The maximum recharge occurred on 4 October, while the minimum recharge was observed on August 6th. Both fixed and semi-active dune watershed plots showed a negative water supply from dunes to lakes on 6 August, indicating a state of water deficit in the system. However, there was a consistent water supply to the lakes throughout the growing season without any shortage in the active dune watershed plots, ensuring continuous water availability. The rainfall distribution determines the temporal dynamics of the water recharge from dunes to lakes, and the vegetation coverage on dunes influences the water quantity that is recharged from dunes to lakes.

To accurately measure the water recharge capacity of dunes to lakes, we used the ratio of the total amount of water recharge to the dune area as a measure of the dune water recharge capacity per square meter from Jun to Oct. The resulting values are presented in Table 3.

There are differences in the water supply capacity of dunes to lakes under different levels of vegetation coverage. The mean water supply capacity decreases from 58.25 mm to 14.87 mm with an increase in vegetation coverage, ranging from active dunes to stabilized dune watershed plots from June to October. The ratio of water supply to precipitation during the same period also decreases from 21.39% to 5.46%. The average water supply capacity of stabilized dune watershed plots is the smallest, and the ratio of replenishment to precipitation over the same period is the lowest. The average water supply capacity of active dune watershed plots is the largest, and the ratio of replenishment to precipitation over the same period is the highest. The water supply capacity of active dune watershed plots is 3.92 times that of stabilized dunes.

#### 3.2.4. Influencing Factors of the External Water Recharge Capacity of Dunes to Lakes

To explore the related factors affecting the water replenishment capacity of dunes, we conducted a Pearson correlation analysis between the water supply from dunes to lakes and precipitation, vegetation coverage of dunes, and temperature. The analysis results showed that the dune water supply to the lake was positively correlated with precipitation, negatively correlated with vegetation coverage, and negatively correlated with temperature (Table 4). The trend of correlation showed that precipitation > vegetation coverage > temperature.

According to the regression analysis (Table 5), the water replenishment of dunes to the lakes was mainly affected by precipitation, vegetation coverage of dunes, and temperature in the watershed.

According to the results of the regression analysis, the regression model is expressed as follows:

Y = 1052.737 + 0.1X_1_ − 11.459X_2_ − 37.785X_3_ (R^2^ = 0.641)

where Y is the water supply of the dune to the lake (m^3^); X_1_ is the replenishment water of precipitation (m^3^); X_2_ is the vegetation coverage of the dunes (%); and X_3_ is the temperature (°C).

Under particular environmental circumstances, we can achieve a water balance in the lake’s watershed by adjusting the dune’s vegetation coverage and the proportion of active dune area in this watershed.

## 4. Discussion

### 4.1. The Effect of Sand-Fixing Vegetation on Lake Water Variation

Previous studies believed that climate change was the main influencing factor of the sand lake [28,29], and they also acknowledged the impact of human sand control projects on sand lakes [30,31]. The conclusion of this study emphasized the influence of artificial sand-fixing vegetation on the redistribution of water resources in the small watershed of the sand lake and clarified the influence of artificial sand-fixing vegetation construction on the sand lake area change. Vegetation influences water dynamics by altering the precipitation redistribution and water consumption. The impact of sand-fixing vegetation on lakes is comparable to the impact of forests on the hydrological processes of river basins in traditional research, and the core of this lies in how vegetation affects watershed hydrological processes. Previous studies have revealed that an increased vegetation cover significantly reduces the runoff yield in forest cover watersheds, especially in arid and semi-arid regions.

In our study, we found a negative correlation between the lake area and vegetation cover from 2000 to 2012, but it was not significant. This may be due to the fact that the vegetation on dunes within the lake watershed remained in its natural state during this period, and the mean rate of vegetation cover change in both plots was minimal during the examined time frame. However, since 2013, with the establishment of artificial sand-fixing vegetation, the vegetation coverage of dunes in the watershed has gradually increased, and its impact on the lakes has also gradually strengthened, showing a significant negative correlation between the vegetation coverage of dunes and the area of the lakes. Therefore, we believe that large-scale artificial sand-fixing vegetation construction on dunes within the watershed will affect the water balance, and with the increase in the dune’s vegetation coverage, the lake area showed a gradual decrease trend.

On the one-year scale, the dunes’ water supply to lakes was negatively correlated with the vegetation coverage. The water supply capacity of dunes with different levels of vegetation cover to the lakes was different. With the increase in sand-fixing vegetation coverage, the external water supply capacity of dunes gradually decreased. During the growing season, active dunes continued to recharge water to lakes, whereas both stabilized sand dunes and semi-active sand dunes did not continue to recharge water to lakes at certain times; the reason for this may be due to the presence of sand-fixing vegetation. Precipitation events only had a short-term impact on the water content of dunes during the growing season, while the presence of vegetation had a longer-term impact on the dunes’ water content. During periods of low precipitation and high vegetation water usage, fixed and semi-active dunes struggled to supply water to the lakes, resulting in a minimal water supply in August. Active dunes can maintain their water supply capacity by having a low vegetation coverage that has minimal impact on the dune’s water storage. Additionally, the development of a dry layer on the surface of the active dunes effectively inhibits soil water evaporation. As a result, active dunes can provide a significant water supply throughout the growing season to the lakes. According to the above research results, by adjusting the vegetation coverage of dunes or increasing the proportion of areas of active dunes in the sand lake watershed, a greater water supply can be provided for the sand lake, which is conducive to improving the stability of the sand lake.

### 4.2. The Practical Significance of the Effect of Sand-Fixing Vegetation on Lakes

Large-scale afforestation aimed at stabilizing dunes may lead to water imbalances in sand lake watersheds, and it may also lead to a lake water reduction or loss in sandy land. This is not beneficial to the sand-fixing vegetation in terms of the long-term development. As a result, many scholars have conducted research on this matter. Yan suggested that active dunes should be preserved in a specific region to collect precipitation and restore groundwater reserves during sand-fixing vegetation construction [32]. Yang et al. proposed constructing a low-density sand-fixing vegetation model, in which vegetation covers 15–25% to maintain its water balance [14]. Alamusa et al. suggested using thinning technology to regulate the density of sand-fixing vegetation and maintain the water balance of sand dunes [21]. This study clarified the impact of dune vegetation on hydrological processes at the watershed scale. A model was constructed to examine the relationship between a dune’s water supply capacity, vegetation cover, precipitation, and temperature within the watershed. The model can be utilized to predict and estimate the recharge of water to lakes in the watersheds. When establishing sand-fixing vegetation in a region, we can refer to the local rainfall and temperature data and maintain the water balance of the lake watershed by adjusting the dune vegetation coverage rate and the proportion of the area of active dunes in the lake watershed. These findings may provide a theoretical basis for the large-scale construction of sand-fixing vegetation.

## 5. Conclusions

On the multi-year scale, when the dune vegetation in the sand lakes’ watershed is in its natural state without any artificial vegetation construction, the changes in the lakes were mainly affected by precipitation. During the process of converting the active dunes to stabilized dunes through artificial sand fixation projects in the lake watershed, as the dune vegetation coverage increased, the area of the lake showed a decreasing trend. The construction of artificial sand-fixing vegetation led to a decrease in the lake area.

On the one-year scale, sand-fixing vegetation affected the water recharge capacity of dunes to lakes in the watershed. As the coverage of sand-fixing vegetation increased, the external water recharge capacity of the dunes to lakes gradually decreased. The dune water replenishment to lakes was significantly positively correlated with precipitation and negatively correlated with dune vegetation coverage and temperature.

A regression model was developed based on the findings of this study, and this model can be utilized to predict and estimate the recharge of water to lakes in the watersheds. These findings may provide a theoretical basis for the construction of sand-fixing vegetation.

## Figures and Tables

**Table 1:** The watershed area of each plot and the area of each landscape component (ha) (2020).

Composition Area	Huituntala	Xigaorisu
Watershed area	508.41	887.21
Inter-dune lowland area	45.50	81.74
Dune area	462.92	805.42
Lake area	8.84	22.54

**Table 2:** Basic conditions of the experimental plots (ha).

Study Plots	T_1_	T_2_	T_3_	L_1_	L_2_	Y_1_	Y_2_	Y_3_
Watershed area	21.02	6.71	64.77	5.99	2.31	13.19	4.47	26.45
Dune area	18.71	4.74	6.09	4.98	1.71	9.18	1.76	19.95
Water area	0.58	0.11	0.19	0.36	0.14	1.12	0.31	1.62

**Table 3:** External water recharge capacity of the dunes to lakes per unit area under different levels of vegetation coverage.

Plot Types	Stabilized Dune				Semi-Active Dune			Active Dune			
Plots	T_1_	T_2_	T_3_	Mean	L_1_	L_2_	Mean	Y_1_	Y_2_	Y_3_	Mean
Replenishment capacity per unit area (mm/m^2^)	15.27	12.57	16.78	14.87	26.17	23.32	24.75	55.70	72.53	46.52	58.25
Ratio of replenishment to precipitation (%)	5.61	4.61	6.16	5.46	9.61	8.56	9.09	20.45	26.63	17.08	21.39

**Table 4:** Correlation analysis of influencing factors of dune water supply to lake. Correlations were either non-significant or significant at the 0.01 (**) or 0.05 (*) level.

	Temperature	Vegetation Coverage	Precipitation Supply
Correlation coefficient	−0.313 *	−0.326 **	0.724 **
Sample size	64	64	64

**Table 5:** Stepwise regression model of influencing factors of dune water supply to lake.

R^2^ = 0.641	Unnormalized Coefficient	SE	Normalization Coefficient	*p* Value	VIF
Constant	1052.737	275.355		<0.001	
Precipitation	0.100	0.012	0.661	<0.001	1.031
Vegetation coverage	−11.459	2.908	−0.302	<0.001	1.029
Temperature	−37.585	11.049	−0.262	0.001	1.041

## Data Availability

Data is contained within the article.

## References

[1] Alamusa, Su Y.H., Yin J.W., Zhou Q.L., Wang Y.C. (2023). Effect of sand-fixing vegetation on the hydrological regulation function of sand dunes and its practical significance. J. Arid Land.

[2] Yin J.W., Alamusa, Su Y.H. (2022). Effects of dune vegetation on water dynamics in inter-dune lowland in the Horqin Sandy Land. J. Desert Res..

[3] Cao J., Alamusa, Zhang Y.H. (2019). Deep Percolation and Lateral Migration of Water in Sandy Dune in the Horqin Sandy Land. J. Desert Res..

[4] Yue D.P., Zhao J.B., Ma Y.D. (2019). Relationship between landform development and lake water recharge in the Badain Jaran Desert, China. Water.

[5] Shao R., Zhang B.Q., Su T.X., Biao L., Cheng L.Y., Xue Y.Y., Yang W.J. (2019). Estimating the increase in regional evaporative water consumption as a result of vegetation restoration over the Loess Plateau, China. J. Geophys. Res.-Atmos..

[6] Yang W.B., Tang J.N., Liang H.R., Dang H.Z., Li W. (2014). Deep soil water infiltration and its dynamic variation in the shifting sandy land of typical deserts in China. Sci. China Earth Sci..

[7] Zhao W.Z., Zhou H., Liu H. (2017). Advances in moisture migration in vadose zone of dry land and recharge effects on groundwater dynamics. Adv. Earth Sci..

[8] Li X.R., Xiao H.L., Zhang J.G. (2004). Long-term ecosystem effects of sand-binding vegetation in the Tengger desert, Northern China. Restor. Ecol..

[9] Kizito F., Dragila M.I., Senè M., Brooks J.R., Cuenca R. (2012). Hydraulic redistribution by two semi-arid shrub species: Implications for Sahelian agro-ecosystems. J. Arid Environ..

[10] Gao J., Kennedy D.M., Konlechner T.M. (2020). Coastal dune mobility over the past century: A global review. Prog. Phys. Geogr. Earth Environ..

[11] Taminskas J., Šimanauskienė R., Linkevičienė R., Volungevičius J., Slavinskienė G., Povilanskas R., Satkūnas J. (2020). Impact of Hydro-Climatic Changes on Coastal Dunes Landscape According to Normalized Difference Vegetation Index (The Case Study of Curonian Spit). Water.

[12] Lichner L., Hallett P.D., Orfanus T., Czarchor H., Rajkai K., Sir M., Tesar M. (2010). Vegetation impact on the hydrology of an aeolian sandy soil in a continental climate. Ecohydrology.

[13] Martínez M.L., Pérez-Maqueo O., Vázquez G., Landgrave R. (2022). Warmer Temperature and Spatiotemporal Dynamics during Primary Succession on Tropical Coastal Dunes. Plants.

[14] Yang W.B., Feng W., Li W. (2016). Principle and mode of combating desertification with low-coverage. Prot. For. Sci. Technol..

[15] Liu X.P., He Y.L., Zhang T.H. (2015). The response of infiltration depth; evaporation, and soil water replenishment to rainfall in mobile dunes in the Horqin Sandy Land, Northern China. Environ. Earth Sci..

[16] Awotwi A., Yeboah F., Kumi M. (2015). Assessing the impact of land cover changes on water balance components of White Volta Basin in West Africa. Water Environ. J..

[17] Hou L., Wang X.S., Hu B.X., Shang J., Wan L. (2016). Experimental and numerical investigations of soil water balance at the hinterland of the Badain Jaran Desert for groundwater recharge estimation. J. Hydrol..

[18] Feng W., Yang W.B., Tang J.N., Li W., Dang H.Z., Liang H.R., Zhang Z.S. (2015). Deep soil water infiltration and its dynamic characteristics in Chinese deserts. J. Desert Res..

[19] Chang X.L., Zhao X.Y., Wang W., Liu L.X. (2013). Response of lake fluctuation to climate change in Horqin Sandy Land. Acta Ecol. Sin..

[20] Carlson Mazur M.L., Wiley M.J., Wilcox D.A. (2014). Estimating evapotranspiration and groundwater flow from water-table fluctuations for a general wetland scenario. Ecohydrology.

[21] Alamusa, Pei T.F., Jiang D.M. (2005). A study on soil moisture content and plantation fitness for artificial sand-fixation forest in Horqin sandy land. Adv. Water Sci..

[22] Huang Z., Liu Y., Qiu K.Y., López-Vicente M., Shen W.B., Wu G.L. (2021). Soil-water deficit in deep soil layers results from the planted forest in a semi-arid sandy land: Implications for sustainable agroforestry water management. Agric. Water Manag..

[23] Kundu S., Khare D., Mondal A. (2017). Past, present and future land use changes and their impact on water balance. J. Environ. Manag..

[24] Zhou G.Y., Wei X.H., Chen X.Z., Zhou P., Liu X.D., Xiao Y., Sun G., Scott D.F., Zhou S.Y., Han L.S. (2015). Global pattern for the effect of climate and land cover on water yield. Nat. Commun..

[25] Bar P., Katz O., Dorman M. (2023). Spatial Heterogeneity Effects on Meta-Community Stability of Annual Plants from a Coastal Dune Ecosystem. Plants.

[26] Descroix L., Laurent J.P., Vauclin M., Amogu O., Boubkraoui S., Ibrahim B., Galle S., Cappelaere B., Bousquet S., Mamadou I. (2012). Experimental evidence of deep infiltration under sandy flats and gullies in the sahel. J. Hydrol..

[27] Ekness P., Randhir T.O. (2015). Effect of climate and land cover changes on watershed runoff: A multivariate assessment for storm water management. J. Geophys. Res. Biogeosci..

[28] Shrestha N., Mittelstet A.R., Gilmore T.E., Zlotnik V., Neale C.M. (2021). Effects of drought on groundwater-fed lake areas in the Nebraska Sand Hills. J. Hydrol. Reg. Stud..

[29] Che X.H., Feng M., Sun Q., Sexton J.O., Channan S., Liu J.P. (2021). The decrease in lake numbers and areas in central asia investigated using a landsat-derived water dataset. Remote Sens..

[30] Zhao Z.L., Liu F.G., Zhang Y.L. (2017). The dynamic response of lakes in the Tuohepingco Basin of the Tibetan Plateau to climate change. Environ. Earth Sci..

[31] Tao S.L., Fang J.Y., Zhao X., Shu Q., Shen H.H., Hu H.F., Tang Z.Y., Wang Z.H., Guo Q.H. (2015). Rapid loss of lakes on the Mongolian Plateau. Proc. Natl. Acad. Sci. USA.

[32] Yan D.R. (2001). Causes and prevention and control of land desertification in Inner Mongolia. Inn. Mong. Environ. Prot..

